# Evolving a Peptide: Library Platforms and Diversification Strategies

**DOI:** 10.3390/ijms21010215

**Published:** 2019-12-27

**Authors:** Krištof Bozovičar, Tomaž Bratkovič

**Affiliations:** Department of Pharmaceutical Biology, Faculty of Pharmacy, University of Ljubljana, Aškerčeva Cesta 7, SI-1000 Ljubljana, Slovenia; kristof.bozovicar@ffa.uni-lj.si

**Keywords:** peptide, combinatorial library, library design, screening, mutagenesis

## Abstract

Peptides are widely used in pharmaceutical industry as active pharmaceutical ingredients, versatile tools in drug discovery, and for drug delivery. They find themselves at the crossroads of small molecules and proteins, possessing favorable tissue penetration and the capability to engage into specific and high-affinity interactions with endogenous receptors. One of the commonly employed approaches in peptide discovery and design is to screen combinatorial libraries, comprising a myriad of peptide variants of either chemical or biological origin. In this review, we focus mainly on recombinant peptide libraries, discussing different platforms for their display or expression, and various diversification strategies for library design. We take a look at well-established technologies as well as new developments and future directions.

## 1. Introduction

Peptides are short polymers composed of 19 l-amino acid and non-chiral glycine residues, linked by amide bonds. The definition is rather vague in terms of chain length, although an arbitrary upper limit of 6000 Da has been assigned to label these molecules peptides, and polymers above that molecular mass are considered proteins [1]. They are ubiquitous in nature and have a role in most physiological processes as host defense (antimicrobial) agents [2], (neuro)hormones [3,4], and toxins [5]. Peptide research has experienced considerable development in the last decades, and over 7000 peptides have been identified in nature [6]. Today, peptides are widely used in drug discovery, drug delivery, food industry, cosmetics, and various other fields.

Peptides and small proteins isolated from natural sources have been used as medicines since the beginning of the 1920s [7], with bovine and pork insulin being the first ones. Transition to synthetic peptide drugs only began in 1950s with synthetic oxytocin and vasopressin entering clinical use subsequently. However, native peptides possess several drawbacks, most notably having poor oral bioavailability and very short plasma half-life, which have tempered enthusiasm for their use, instigating investigators to develop peptides with improved pharmaceutical properties [8]. But it would be one of the biggest breakthroughs in understanding the fundamentals of life itself, the discovery of the genetic code and how it translates to the amino-acid sequence, laying foundations for modern biotechnology, which really pushed the field in a whole new direction in the coming decades. Gene identification and manipulation techniques that developed rapidly from 1973 onwards allowed for producing large quantities of pure gene products [9]. The landmark event was the expression of the first recombinant peptide hormone insulin in *Escherichia coli* and the following approval to commercialize recombinant insulin in 1982.

Peptides are utilized broadly owing to their superiority in specific cellular targeting. They bind cellular receptors with high potency and great selectivity, lowering toxicity potential and occurrence of off-target effects. In addition, peptides in the body are degraded to amino acids, further lowering the risk of toxicity [10]. Chemical synthesis enables peptide fabrication in large quantities, chipping production costs compared to other biologics. More attributes include stability at room temperature and good tissue permeability. Furthermore, physico-chemical traits of peptides (e.g., solubility, hydrophobicity, and charge), metabolic stability, and their residential time in the body can be fine-tuned through chemical modifications. Reiterative chemical modification approach can be honed for development of peptide therapeutics with improved properties [11], including extraordinary target affinity [12]. 

Areas of the highest concentration of peptide development in medicine are metabolic diseases, oncology, and cardiovascular diseases, not surprisingly, all areas of highest interest to the pharmaceutical industry. By 2018, more than 60 peptide drugs (excluding insulins and other small proteins) have been approved in the US, Europe, and Japan, over 150 were in active clinical development, and an additional 260 were assessed in human clinical trials but did not make it to the market [8]. The peptide therapeutics market was valued at 19,475 million USD in 2015 and it is estimated it will more than double the value by 2024, reaching 45,542 million USD [13].

During the past decade, peptides have also been used in a wide range of applications in other fields. They are found in biosensor applications as biorecognition molecules and are conjugated with transducers or molecular beacons that aid signal detection [14,15]. Additionally, they serve as surfactants or tags promoting solubility of recombinant intrinsic membrane proteins [16,17,18,19,20], increasing their yield, activity, and aiding protein structural studies. Peptides are even replacing enzymes in catalytic reactions [21] and substituting proteins as ligands in affinity chromatography [22,23].

Discovery and design of novel peptides can be guided by various strategies. In this review, we focus mainly on the use of peptide and peptide aptamer [24] (sequences of 5–20 amino acid residues, grafted into loops of a robust protein scaffold) libraries generated through recombinant DNA technology, but discuss chemical peptide libraries as well.

## 2. Combinatorial Peptide Libraries

Peptides of great number and diversity occur as a natural form of combinatorial chemistry. Conversely, exploiting evolutionary principles in the laboratory by constructing and screening large peptide libraries can yield new lead compounds with desired traits. The discovery of novel binders is a multifaceted process involving scanning of thousands or even millions of potential candidates from combinatorial libraries using in vitro screening analysis, commonly used in target-based drug discovery. Target-based drug discovery (sometimes called “reverse pharmacology”) is the opposite of a traditional phenotypic screening strategy. The latter typically leads to the identification of molecules that modify a disease phenotype by acting on previously unidentified target [25]. In contrast, the targets in the target-based approach are well defined. With the molecular target in hand, discovery of novel binders can be facilitated by utilizing crystallographic and biochemical studies, computational modeling, binding kinetics, and mutational analysis to gain insight into how the target and the ligand interact and thus enable efficient structure-activity (SAR) analysis and the development of future generations of binders [26].

Combinatorial peptide libraries can be categorized into two groups—chemical peptide libraries, which are produced via organic synthesis, and biological libraries. Choosing a library platform should be guided by practical manners. Importance of library size, the experience of operators, available equipment, and other technical considerations may well limit the choice [27]. In principle, library-based peptide discovery adheres to the following paradigm: (1) creation of a pooled peptide library, (2) screening of the library against the target molecule and isolation of hits, and (3) hit identification.

Various screening/selection methods are at disposal depending on the peptide library platform. Normally, screening peptide libraries involves incubating the library with a fluorescently labeled soluble target or target-coated magnetic beads, followed by flow cytometry-based systems such as fluorescence activated cell sorting (FACS) [28], or magnetic separation techniques like magnetic-activated cell sorting (MACS) [29], respectively. The former is mostly used for cell-based peptide libraries, although it has also been used for screening chemical library systems such as one-bead-one-compound platform [30] (see below). Hit identification is also dependent on the library type; either iterative deconvolution or positional scanning methods are used for chemical libraries, while sequencing is typically employed for DNA-encoded platforms. In recent years, next-generation sequencing (NGS) methods, capable of massively parallel nucleic acid sequence determination, have transformed the field of screening biological libraries, enabling detection of low abundant clones and quantification of changes in clone copy numbers without performing many rounds of selection. This technology has been paired with various library platforms, immensely enhancing the throughput of these methods [31,32,33,34,35,36,37,38].

### 2.1. Chemical Peptide Libraries

In chemical approach, the library synthesis is either completed on a solid support (insoluble porous polymeric resin) and then members are cleaved to be screened as free compounds, or the library is synthesized and screened on a solid matrix.

Solid-phase peptide synthesis (SPPS) was first achieved by the Nobel laureate Robert B. Merrifield. The general approach of SPPS is to attach the first amino acid to a solid support through its carboxyl group. Subsequently, each N-protected amino acid is then added in turns. During coupling, the carboxyl group of the incoming amino acid must be activated, which is commonly achieved by using carbodiimides, amino acid halides, uronium (guanidinium N-oxides), or phosphonium salts [39,40]. After each addition, the N-protecting group must be removed before the next amino acid is added. The most used N-protecting group is the fluorenylmethyloxycarbonyl (Fmoc) group [41] which can be orthogonally removed under basic conditions. Since the growing peptide chain is attached to a surface, removing waste products of synthesis is accomplished by simple washing. Combinatorial synthesis is inherently a parallel synthetic process where a single product is obtained in each different reaction flask (or a sealed permeable container (“teabag”) submerged in a reaction flask) [42]. Alternatively, a mixture of products can be obtained in a single flask via the “mix-and-split” method (see below) [43]. The whole synthesis process is quantitative, as the reactions are driven to completion by applying reagents in excess at every step. In addition, as the growing peptide chain is attached to a matrix, there is no need for isolation of intermediates. Nevertheless, judicious use of resources should be considered as wasting large amounts of reagents is costly and the surplus effluent produced can be an environmental burden [1], although this is a concern mainly when producing large quantities of product and less so for library generation.

**Figure 1:**
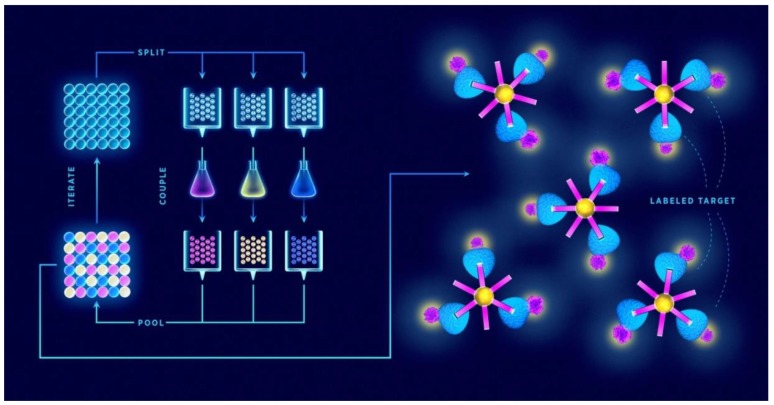
Synthesis of combinatorial peptide libraries by the “split-and-mix” method. Carrier beads are split into aliquots (only 3 are shown for clarity) for coupling individual amino acid residues, pooled, and the process is iterated until the desired length of peptides is achieved. Library diversity increases exponentially with each coupling step. At the end, each bead carries a single peptide sequence; hence, the name “one-bead-one-compound” combinatorial library. Libraries are typically screened by incubating the beads with a fluorophore-labeled target and subsequent fluorescence-activated sorting.

For generating large libraries, the “split-and-mix” is the preferred strategy. This cyclic scheme of SPSS was first demonstrated by Furka et al. [43] in 1991 and involves coupling of individual amino acids to resin beads, mixing the beads together, separating them in equal portions, and then reacting each portion with a different amino acid (Figure 1). Mixing, separating beads, and the reaction step are repeated until the desired peptide length and diversity are achieved. An important virtue of this method is that a single bead contains a single peptide sequence, which is why the libraries produced in this way are termed one-bead-one-compound (OBOC) [44]. The second strategy is called “pre-mix”, where all the amino acids to be coupled at each synthetic step are premixed in equimolar ratio and then reacted with a single resin batch. To overcome the problem of different kinetics rate of each amino acid coupling, “smart” mixtures of molar ratios that correspond to different coupling rates were proposed [45].

Various deconvolution methods have been developed for screening and identification of hits from chemical peptide libraries; most commonly adopted are iterative deconvolution [46] and positional scanning [47]. Iterative deconvolution is based on dividing the library into non-overlapping subsets containing peptides with defined residues at the specified position(s) (while having the rest of the structure randomized). Each subset is then screened separately. The most active subset of compounds is further divided into new subsets (retaining the identified optimal residue(s) from previous screening round) and retested for activity. This process is continued until the fittest molecule is identified [42]. In positional scanning, sub-libraries individually address each diversity position. This position is defined with a single amino acid residue, while the remaining positions are fully randomized. Positional sub-libraries are assayed in parallel to gather information on the optimal residue for each diversity position, consequently identifying the fittest member [47]. Other hit identification techniques include Edman degradation [48] and mass spectrometry (MS)-based strategies [49,50]. Mass spectrometry is highly sensitive and very fast, and is the method of choice when it comes to generating sequence data in the femtomolar range. Edman degradation, although reliable, only becomes a viable option at picomolar quantities [51]. It is also slow and not easily amenable to multiplexing [52]. In contrast, mass spectrometry has the edge in analyzing mixtures of great diversity, making HPLC separation of peptides unnecessary. However, a complete peptide fragmentation pattern is required in order to unambiguously identify each amino acid sequence [50].

The cutting-edge technology in chemical combinatorial libraries is the so-called DNA-encoded library (DEL) platform. It works by tagging the library members via an adapter module to a double-stranded DNA barcode which enables the unambiguous identification of retained compounds at the end of the selection process [53]. Both the library synthesis and tagging rely on the “split-and-mix” approach. In each synthesis cycle, the process is encoded (recorded) by ligation of a short DNA tag that identifies the amino acid residue added. A pooled library is then assayed by affinity selection and selected binders can be easily identified by PCR amplification and sequencing of tags [54,55]. Some peptides identified using the DEL platform include carbonic anhydrase binders [56], ligands of integrins, and CCR6 [57]. Generation of macrocyclic peptide libraries by DEL has also been described [55,58]. DEL offers several benefits over some other established methods, like phage display (see below), for discovery of peptide ligands. Library sizes (i.e., diversity) are larger, allowing the chemical space to be more deeply sampled in a single screen. Furthermore, DEL platforms are compatible with parallel screening against multiple targets, enable activity-agnostic screening for identification of “silent binders”, and consume relatively low amounts of reagents [54]. In addition, not only individual screening hits are identified, but rather, larger families can be detected, in which related building blocks or combinations thereof are enriched [59]. Limitations of the platform include possible interference of the oligonucleotide tag in target–binder interaction that may cause steric hindrance. The barcode can also restrict the extent of possible chemical reactions and influence the properties of binders. Resynthesis after selection must be performed for subsequent target binding validation, which adds to the duration of the assay [54,60].

### 2.2. Biological Peptide Libraries

A key feature of biological libraries is the linkage of the genotype (i.e., genetic information) with its corresponding phenotype (the encoded peptide/protein). In directed evolution, (random) mutagenesis (gene diversification) and screening for functional gene products are iteratively alternated, and the phenotype:genotype link is crucial for peptide identification via sequence determination of the encoding oligonucleotide. Based on the display type approach, biological libraries can be categorized as either cellular or acellular [61].

#### 2.2.1. Cellular Approach

**Figure 2:**
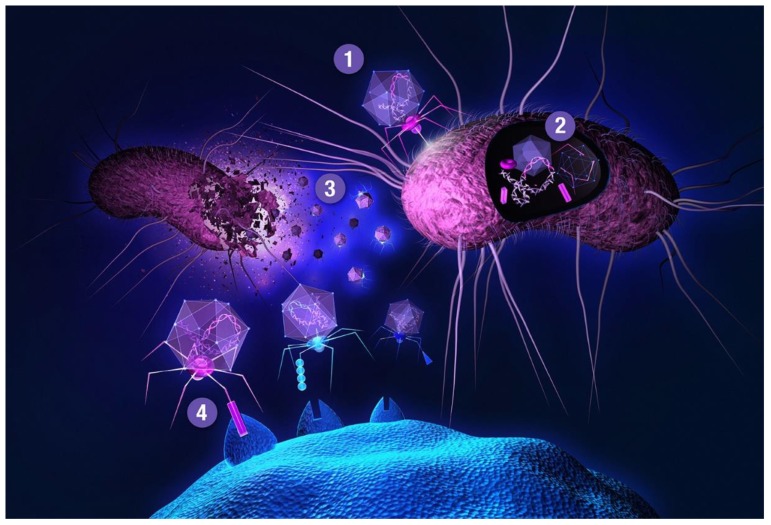
Iterative principle of phage display library screening. Recombinant phage DNA is packed into viral particles in vitro (common for T7- and lambda phage-based systems) for transduction of genetic library into host bacteria (**1**). Alternatively, phage DNA can be electroporated into host cells (typical for filamentous phage-based systems; not shown). Exploiting bacterial transcription and translation mechanisms, progeny virions displaying foreign peptides are amplified and assembled (**2**), and released into growth medium (**3**). Amplified library is isolated and purified, and contacted with an immobilized target (**4**). Non-bound clones are removed by stringent washing, while those retained due to target:displayed peptide interaction are eluted and collected for amplification in host bacteria before being subjected to further selection rounds.

The main bottleneck to this approach is a transformation step needed for delivering a DNA library into host cells, providing transcriptional and translational machineries for gene expression. One of the most widely used and recognized methods is phage display, first described in 1985 by George P. Smith [62]. Smith shared half of the 2018 Nobel Prize in chemistry (the other half was awarded to Frances H. Arnold) with Gregory P. Winter for the phage display of peptides and antibodies, validating the gargantuan importance of this technology. Filamentous (bacterio)phages (most commonly used vehicles in phage display) are rod-shaped viruses that infect *E. coli*, and are composed of coat (capsid) proteins that encapsulate the phage genome. Surface display is achieved by inserting a peptide-encoding oligonucleotide sequence into one of the genes for a capsid structural protein [63,64,65]. All 5 filamentous phage coat proteins have been exploited as anchors for display of foreign peptides and proteins. The filamentous phage’s minor coat protein p3 is the most widely used and can present 3–5 copies per virion, seconded by the major coat protein p8 with a much larger count, around 2700 copies. The filamentous phage M13 and the closely related fd are most commonly used due to their ease of manipulation and their ability to accommodate fairly large pieces of foreign DNA. Two main display types emerged for displaying libraries on filamentous phage: the polyvalent (one-gene-system) and the monovalent display (two-gene-system). In the polyvalent display, the DNA fragments are inserted into the phage vector, producing fusions with each copy of the chosen coat protein [65]. As opposed to monovalent display, polyvalent systems yield binders that exhibit reduced affinity due to avidity effects, enriching weak (albeit specific) ligands over the course of a multi-step affinity selection process [66]. The monovalent display uses a phagemid vector—essentially a plasmid encoding the foreign peptide-coat protein fusion, and harboring the phage origin of replication and the packaging signal for production of single-stranded DNA copies and assembly into phage particles, respectively. A helper phage (which supplies the rest of phage genes, but has assembly defects) is employed to superinfect the cells harboring the phagemid. Thus, the produced virions display a mixture of recombinant coat proteins, encoded by the phagemid, and the cognate wild-type proteins, encoded by the helper phage [27]. Although two-gene-systems are mainly utilized for displaying larger proteins, both systems have been used to display relatively short peptides [67]. Less commonly, lytic phages, such as T7 or lambda, have been used for phage display of peptides [64,68]. Another interesting phage display-like system allowing tunable display valency is based on virus-like particles (VLPs) of the RNA bacteriophage MS2 [69]. In phage display, target-binding peptides are identified through affinity selection process called panning (Figure 2). This technique is comprised of several steps. First, surface-immobilized target is contacted with phage library, followed by stringent washing to remove unbound and non-specifically bound clones. Specific binders are then eluted, usually by applying pH shock or high salt solutions, and subsequently amplified in host cells. Obtaining binders with high affinity usually requires 3–5 panning rounds. There is a reason why phage display is the method of choice for so many. Phage libraries are more readily affordable compared to using other microbial/cell display vehicles (see below), can easily be amplified by allowing library phage to replicate in a bacterial host. Virions can withstand various selection environments, endure harsh washing and elution conditions, and can be stored at –80 °C for years. Phage library diversities are typically significantly larger compared to chemical peptide libraries and can reach up to 10^11^ clones. Although phage can accommodate a large variety of (poly)peptide structures, amino acid and sequence biases do occur (as with any biological library), leading to inherent library diversity decrease [70]. In filamentous phage, this is due to censorship of charged sequences through the general bacterial secretory (Sec) pathway. Capsid structural proteins fused to charged peptides seem be to less efficiently inserted into the *E. coli* inner membrane, hindering virion assembly [71]. Conversely, lytic phage vehicles have the advantage of not being limited to display of peptides which are efficiently translocated to the periplasm [72]. To display (poly)peptides that fold rapidly in the cytoplasm (and are thus inefficiently transported to periplasm) on *filamentous* phage, the twin-arginine translocase (Tat) export pathway has been exploited instead of the conventional Sec pathway [73,74]. In contrast to Sec translocase complex that mediates export of unfolded proteins through the inner bacterial membrane, Tat only exports fully folded proteins. Another drawback of phage display is the non-quantitative nature of clone screening, although this problem can be elegantly tackled by deep sequencing [31] instead of traditional clone picking. In conventional phage display, the displayed peptides are limited to natural L-amino acids. All-D peptide ligands, showing improved metabolic stability, can be developed using the mirror-image phage display strategy [75]. Cyclization of phage-displayed peptides [76] represents another popular method of augmenting protease resistance and simultaneously increasing affinity (also see Section 3). In addition to in vitro or ex vivo (e.g., against isolated cells), phage library selections can also be performed *in vivo*. The latter approach is used to identify tissue homing peptides for cell-specific targeting of therapeutics [77]. In contrast to phage library pannings to purified targets, selections against intact cells and in vivo screens are inherently more complicated as the library is exposed to many potential decoys, leading to high background [63]. When panning against a cell surface receptor, one typically relies on a cell line ectopically overexpressing the targeted membrane protein. This allows for so called *subtractive* or *negative* selections to be performed against the same cells devoid of the target of interest before contacting the library phages with target-transfected cells in each selection round, thereby effectively reducing background binders. In in vivo selections, phage library is typically injected intravenously or directly into tumor xenografts of an experimental animal. Upon systemic application, library phages are likely not distributed uniformly to all tissues, but are rather retained in the circulation, being primarily exposed to vascular endothelium. Targeting other tissues requires fairly large phage titers; phages can be rescued from tissue of interest, and amplified for iteration of selection. Deep sequencing enables analysis of the entire repertoire of retained phages [78], and should thereby allow the deduction of specific binders even from single-selection-round experiments [79], especially if independent parallel screens are performed and the same peptides are observed enriched in each case. To limit background phages, vascular perfusion can be harnessed.

Several peptide display systems based on Gram-negative bacteria were reported. Typically, peptides are grafted in the surface-exposed loops of outer membrane proteins. Fusion partners for peptide library display include the *E. coli* OmpA [80], OmpX [81], and the *Pseudomonas* OprF [82] among others. It is also possible to display peptides on extracellular appendages such as pili [83] and flagella, albeit only the latter has been used for library construction [84,85]. Conventional bacterial surface display libraries are commonly screened using FACS. Library display and selection have also been demonstrated in the periplasm of *E. coli*. In the intra periplasm secretion and selection (PERISS) system, the target molecule gene and peptide library DNA are integrated in tandem into a plasmid, expressing the peptide library in the periplasmic space, while the target molecule is incorporated into the *E. coli* inner membrane. The outer membrane is disrupted with ethylenediaminetetraacetic acid (EDTA) and the cell wall is degraded enzymatically to form spheroplasts. Peptides interacting with the target are collected as binding complexes on magnetic beads through a fusion tag, and identified through PCR amplification and sequencing [86]. Another form of periplasmic display is the “anchored periplasmic expression” (APEx) technique, where a peptide library is expressed and immobilized in the periplasm of *E. coli* via fusion to a lipoprotein targeting motif that enables anchoring to the inner membrane. The outer membrane and the cell wall are then removed, followed by incubation with the fluorescently tagged target molecule, washing, and binder detection by FACS [87,88]. Furthermore, peptide libraries were expressed in the cytoplasm of bacteria via fusions to the C-terminus of the *lac* repressor. The repressor protein binds to *lac* operator sequence on the plasmid encoding the peptide, providing a physical genotype-phenotype link. The library is screened by affinity purification with an immobilized receptor [89]. Screening techniques of the latter are inferior compared to surface display, as the cells must be lysed prior to panning to the immobilized target. Another important technique, the bacterial two-hybrid system, is used for studying protein–protein interaction (PPI). It can be used to screen libraries of peptides to probe and manipulate biological pathways [90]. The premise of all variants of two-hybrid platforms is the identification of target binders via reconstitution of reporter’s activity in vivo dependent on the interaction between a pair of mediator proteins. Typically, the target protein (“bait”) is fused to the transcriptional activator’s DNA-binding domain (interacting specifically with the upstream activating sequence of a reporter gene), while the library (poly)peptide variants (“prey”) are fused to the transcriptional activation domain (which recruits the RNA polymerase). Another option is the functional complementation of a split two-domain enzyme adenylate cyclase upon bait-prey interaction, leading to indirect reporter gene activation [91]. Interactions between protein pairs are identified by growing library bacteria either on color indicator or selective media plates [92]. In general, assets of bacterial combinatorial libraries are fast bacterial growth rate, ease of genetic and physical manipulation, and (in case of surface display and periplasmic expression) highly efficient screening protocols based on FACS. On the other hand, display platforms may suffer from interference of the complex bacterial surface with the selective peptide:target recognition. A recently developed peptide library system termed “surface localized antimicrobial display” (SLAY) [93] relies on a “reversed” screening protocol to identify peptides with antimicrobial activity. In SLAY, library plasmids are transformed into bacteria of interest and induced to express the encoded peptides. The peptides with antibacterial properties will lead to bactericidal or bacteriostatic effects, eliminating these clones from the population. Using NGS and in silico techniques, sequences pre- and post-induction are analyzed to identify members with antimicrobial properties.

Phage and bacterial display can be classified as prokaryotic display. There are also vast possibilities of displaying peptides and proteins in eukaryotic systems. Their main advantage is the reliable folding and glycosylation of eukaryotic proteins [94]. One of the most widely used organisms in this category is *Saccharomyces cerevisiae*. Yeast has been utilized as a vessel for numerous types of peptide library screening. Like the bacterial two-hybrid system, the yeast two-hybrid system is used for investigating protein–protein interaction through the activation of reporter genes responding to a reconstituted transcription factor [95,96]. This particular method gave rise to a number of adaptations to study PPIs, such as the *ras* recruitment system [97], split ubiquitin system [98], and the yeast three-hybrid system [99], to name a few. Conversely, yeast surface display is achieved by fusions to a cell surface-anchored protein, followed by screening and selection through magnetic separation or FACS [100]. Foreign peptides are commonly fused to the C-terminus of α-agglutinin Aga2p subunit, a surface protein covalently bound to glucan, which mediates cell to cell adhesion during yeast mating. Aga2p is linked to the Aga1 protein through two disulfide bridges, resulting in a covalent complex on the surface of the yeast cell [101]. Other surface anchor proteins used in this manner are Agα1p, Cwp1p, Cwp2p, Tip1p, Flo1p, Sed1p, YCR89w, and Tir1, a choice that depends on the type of protein/peptide to be displayed [102,103]. Another type of display in yeast is possible via secretory expression [104]. A number of novel ligands have been discovered via screening of peptide or small protein libraries expressed in yeast, both on the surface and intracellularly. Examples of surface display libraries include cysteine knot peptides (knottins) [105] and lanthipeptides [106], whereas head-to-tail cyclized peptide libraries [96,107] (see the SICLOPPS method description below) are expressed intracellularly. The main advantage of yeast display is its eukaryotic protein expression mechanism, which allows for complex post-translational modifications, and quantitative library screening through FACS [108,109]. Disadvantages include smaller library sizes due to low transformation efficiency [110], and lower affinity caused by unintended multivalent binding to oligomeric targets, although this can be surmounted by applying kinetic selections [111]. Yeast also form high-mannose type glycans that render glycoproteins produced in this system unfit for human applications, but this problem can be tackled by humanizing yeast glycosylation pathways [112].

Another platform well-suited for (poly)peptide display is the baculoviral particle (or baculovirus infected cell) system. Baculoviruses, enveloped viruses infecting invertebrates, are distinguished by a large packaging capacity, and (being eukaryotic pathogens) support diverse post-translational modifications [113]. Typically, foreign peptides or proteins are fused with the major envelope glycoprotein, such as the gp64 of the baculovirus *Autographa californica* multiple nuclear polyhedrosis virus (AcMNPV), and the recombinant fusions are embedded in the viral envelope (and the plasma membrane of infected insect cells) along with the wild-type glycoprotein. Other membrane anchoring strategies can be exploited to display a wide range of proteins and peptides at diverse valency range [114,115]. Regardless of the display strategy, libraries on infected insect cells are screened with FACS [116], and libraries displayed on the AcMNPV virions may be selected via conventional affinity panning [117]. In a prominent example, baculovirus-displayed libraries of peptides bound to the class I or II major histocompatibility complex (MHC) have served for identification of T cellular receptor peptide antigen mimetics (mimotopes) [118,119]. Combining the benefits characteristic for prokaryotic platforms (e.g., simple viral propagation as insect cells do not require CO_2_ exchange for growth, high transfection rates, and the low-risk biosafety profile [114,120]) with clear eukaryotic advantages (efficient protein folding and a plethora of accessible post-translational modifications), the baculoviral display platform seems underappreciated. On the other hand, downsides of using this technology are expensive growth media [121], similar but still different glycosylation patterns compared to those of mammalian systems [122] and time-consuming cloning in the baculoviral vector, although new systems to avert this lengthy step have been developed (reviewed by Possee and King [123]).

Peptide libraries have also been displayed by using several eukaryotic RNA viruses with inserting short peptides into their native envelope proteins without interfering with the viral infectivity. Examples of eukaryotic retroviral vehicles include the avian leukosis virus [124] and feline leukemia virus [125,126]. Alongside retroviruses, the adeno-associated virus (AAV) has been used as a platform for peptide library display. Here, capsid diversification combined with phenotypic screening was primarily used as a means of achieving re-targeted tropism [34,127,128,129,130,131]. Normally, AAV libraries are produced in human cells, such as HEK 293T or HeLa [132]. AAV library construction starts by inserting encoded capsid gene variants (in place of previously excised wild-type *cap* gene sequence) in a template vector encoding a complete AAV genome. Because the natural vector tropism is disrupted, the rare subpopulation of library peptides are expected to redirect virions to new, formerly inaccessible cell types [133]. Library screening involves passaging the virions over several rounds in the chosen cell type to enrich for capsid variants with higher transduction efficiency and/or specificity compared to the naïve library background [134]. AAV libraries are primarily considered for their modus operandi—they infect humans and can thus be exploited as target-specific gene therapy vectors. AAV does not seem to cause any diseases and is only weakly immunogenic. Many challenges face AAV library platform, most notable being an uncertainty of capsid-genome correlation due to the possibility of co-transfection with different AAV capsid variants, resulting in library members with multiple phenotypes [132].

Peptide libraries have also been displayed on the surface of mammalian cells. Examples include fusions to the chemokine receptor CCR5 for antibody mimotope selection [135] and cystine-dense peptides for difficult to drug targets [36]. On the other hand, peptide libraries have been deployed for intracellular screens in mammalian cells [136,137,138,139]. Besides the benefit of authentic post-translational modifications, the most conspicuous advantage of mammalian display libraries is the ability to screen against targets and to investigate PPIs in their native environment. On the other hand, obvious downsides of working with mammalian cells are high cultivation costs and laborious cultivation technologies.

The “split-intein circular ligation of peptides and proteins” (SICLOPPS) is a method for cyclic peptide library generation. It takes advantage of intein splicing to generate peptide libraries in cells [140]. Inteins are self-excising protein domains that process to link their consecutive sequences with a peptide bond, while liberating the intervening portions (exteins) as head-to-tail cyclized peptides [141]. Library peptides to be cyclized are usually 6 amino acids long randomized exteins, flanked by C- and N-terminal intein domains with splice site-adjacent cysteine residues. Propagation of plasmids harboring SICLOPPS expression cassettes in cells resolves the problem of genotype:phenotype linkage. As the libraries are assembled in cells, this approach is particularly well-suited for functional assays; both function and affinity (to a large repertoire of accessible intracellular targets) for each binder can be assessed. Other benefits include simplicity, speed, and ease, and its applicability to different organisms [107,142,143] make this high-throughput approach the platform of choice. On the other hand, the main bottleneck of SICLOPPS is the limited library size of 10^7-9^ (determined by transfection efficiency) [140,144,145].

#### 2.2.2. Acellular Approach

In contrast to the cellular approach, the acellular libraries are not propagated in living cells. These systems are based on in vitro transcription and translation, and one of the main advantages of this approach is the potential to generate high diversity libraries with up to 10^15^ individual entities by averting cell transformation step. Also convenient is the ability to apply stringent screening conditions that would be incompatible with cell viability.

In ribosome display, peptide:ribosome:mRNA complexes are generated, physically associating nascent peptide to their encoding mRNA. This is achieved by eliminating stop codons, consequently stalling both the nascent peptide and its encoding mRNA on the ribosome. The method consists of the following steps: DNA library preparation, in vitro transcription and translation, affinity selection and mRNA recovery, followed by reverse transcription and PCR amplification for sequencing or further selection rounds [146]. Many types of peptide libraries have been screened through this platform, including tumor targeting peptides [147], peptides that bind to monoclonal antibodies [148,149], streptavidin ligands [150], and metal binding peptides [151], to name a few. Besides its simplicity and the obvious ability to create high diversity libraries, another key advantage of the technology is that the diversity of the libraries can be easily manipulated by introducing new mutations at any selection step, and is therefore particularly suited for directed evolution projects [152]. With the advances such as PURE (protein synthesis using recombinant elements), ribosome display has also become very stable, as it is composed only of reconstituted ribosome and translation factors, eliminating degradation of mRNA and nascent (poly)peptides by endonucleases and proteases from the cell extract [153]. Another detriment that is being resolved is the thermal stability of this platform; usually, ribosome display is performed at near freezing temperatures. Fusing the library peptides to the Cv RNA-associating protein (Cvap) and adding the Cv RNA motif to the 5’ mRNA end renders the peptide-Cvap:mRNA:ribosome complexes stable at room temperature, even for prolonged time [151,154]. However, the fragile noncovalent phenotype:genotype conjugation still requires mild selection conditions.

Another technique, mRNA display, is similar to ribosome display. The essence of this technology is the employment of puromycin, an antimicrobial product that inhibits translation by mimicking the 3’-end of an aminoacyl-tRNA, conjugated to the 3’ terminus of mRNA [155]. When the ribosome reaches the 3’ end of the template, the newly synthesized peptide is transferred onto the puromycin, achieving genotype–phenotype linkage with its encoding mRNA. Later, affinity panning is the preferred practice for binder identification. As in ribosome display, after each selection round, mRNA is reverse transcribed to cDNA and PCR amplified for the purpose of sequencing or further selection rounds. This platform has been extensively used and there are numerous examples of discovered novel peptide binders. These include cyclic peptide therapeutics targeting GPCR signaling [12], streptavidin binding peptides [156], and peptide vaccines [157]. Compared to ribosome display, this platform is superior and enables stringent selection conditions [158]. A major advantage over ribosome display is obviously the absence of the ribosome, a huge 2,000,000 Da ribonucleoprotein complex, which can interfere with the selection process [159]. Despite its unique ability in addressing a repertoire of different biological problems, mRNA display has limitations like any other display technology. One of the concerns involves interactions of covalently bound mRNA with the displayed peptide or the target molecule. The interference of flexible mRNA is particularly problematic when dealing with proteins that nonspecifically bind nucleic acids [158]. Furthermore, the highly negatively charged mRNA fusion moiety may interfere with positively charged target molecules [160].

A major problem in both ribosome and mRNA display is mRNA lability. This was the driving motivation behind the development of the cDNA display method. Here, mRNA is ligated to a looped DNA linker, harboring a primer region for reverse transcription and a restrictase cleavage site, and carrying a loop-attached biotin group and a 3’ terminal puromycin moiety. Thus, the translated mRNA-encoded peptide is transferred to the DNA linker, and the complexes are captured on streptavidin beads for reverse transcription. Finally, the library is released from the solid support by restrictase treatment [161]. This refined platform has been used in screening for cysteine-rich peptides with antagonistic activity at interleukin-6-receptor [162], peptide antagonists of growth hormone secretagogue receptor [163], amino group binders [164], binders of vacuolating toxin [165], and peptide agonist and antagonist of angiotensin II type-1 receptor [166]. The problem of RNA instability instigated the design of another DNA-based approach called CIS display. This method harnesses the so-called cis activity—the capacity of a DNA replication initiator protein (RepA), fused to the library peptides, to bind exclusively to the cognate template DNA [167]. CIS display has been applied for identification of binders of antibodies [167], protease-resistant peptides [168], and ligands of human vascular endothelial growth factor receptor [169].

Another acellular platform dubbed “in vitro compartmentalization” (IVC) mimics natural encapsulation of living cells by entrapping a DNA library and a transcription/translation reaction mixture in water-in-oil emulsion droplets. These “microreactors” serve as boundary analogs to a cell membrane, assuring an effective genotype–phenotype linkage; on average, each droplet contains a maximum of one library member. The in vitro transcription/translation paraphernalia within the droplets processes the genes, and the desired phenotype is selected using a suitable strategy [170]. For example, the DNA can be linked to a substrate which is converted into a marker product by the encoded enzyme, rendering it detectable by FACS [171]. It has been reported, that 1 mL of such an emulsion can hold as many as 10^10^ droplets [170]. Adaptations of this technique (e.g., STABLE [172], SNAP [173]) have been summarized in a recent review [174]. Although IVC is a technology especially handy for in vitro enzyme evolution [175], it has also been applied for screening peptide libraries [176,177]. Possessing benefits of the above acellular platforms, this technology is superior as reactions can be controlled inside the droplets, by adding reaction constituents in a step-wise fashion at defined time-points (e.g., after in vitro translation) [178]. IVC is also suitable for quantitative screening using FACS [179]. There are technical limitations to this platform, and the one that stands out is the formation of double emulsion droplets with the consequence of severing the genotype–phenotype link, although this could be solved by employing a high-throughput screening platform using droplet-based microfluidics [180]. IVC libraries are also considerably smaller than mRNA-display libraries [181].

## 3. Incorporating Unnatural Amino Acids and Constraints into (Library) Peptides

The methods described above have become invaluable for the discovery or refinement of peptide binders. Still, low-molecular-weight leads are generally preferred over peptides in drug discovery, owing to peptides’ inadequate properties. For example, libraries of natural peptides are limited to the 20 proteinogenic amino acids and are normally restricted to linear peptides that have high flexibility and poor pharmacokinetic properties. The natural amino acid limit makes it difficult (if not impossible) to introduce predefined pharmacophores into peptide libraries. Moreover, the exploration of “chemical space” is limited by the restricted set of residues, many of which share the same or similar side-chain chemical groups. Marked flexibility of the peptide backbone imposes high entropic cost upon peptide ligand binding to the target molecule, resulting in low affinity interactions [182,183]. Linear peptides can also fall prey to enzymatic cleavage by exoproteases [184]. Potent and proteolytically stable binders can be designed by introducing modifications into peptides, such as incorporation of non-proteinogenic residues [185], cyclization [186,187], or the inclusion of stabilizing moieties (e.g., the parallel β-sheet scaffolds) [188]. Another option is the introduction of chemical post-translational modifications (cPTM) that can vastly expand the diversity of peptide libraries, for example, by phosphorylation [189], conjugation to glycans [190,191], attachment of a fluorescent reporter [192], or ligation to a synthetic peptide fragment [193]. By introducing constrained topologies, cyclic [194] or bicyclic [195] variants can be generated [187]. However, cPTMs are not quantitative. These moieties are also not genetically encoded, and thus cannot be identified via sequencing, although this problem can be overcome with the construction of chemically identical peptide libraries using different codons (“silent barcodes”), signifying a specific cPTM [196].

Genetic code “reprogramming” refers to reassignment of arbitrary codons from proteinogenic to non-proteinogenic amino acids, allowing ribosomal synthesis of non-canonical peptides. This strategy is compatible with acellular in vitro display techniques. By omitting aminoacyl-tRNA synthetases (ARSs), library diversity can be increased by utilizing a reconstituted translation system such as PURE [153]. tRNAs can be charged with nonstandard or artificial amino acids employing natural or modified ARSs [197]. Promiscuous tRNA acylation ribozymes, termed “flexizymes”, were created as a surrogate for ARSs. Flexizymes recognize activated carboxylates and allow extensive genetic code reprogramming [198]. Combining flexizymes with a custom in vitro translation system, a new technology, dubbed FIT (flexible in vitro translation), was developed. Adding mRNA display to the mixture, the so-called RaPID (random non-standard peptide integrated discovery) system was born, facilitating the discovery of potent nonstandard peptides for therapeutic and diagnostic use [38].

## 4. Peptide Library Design and Construction

**Figure 3:**
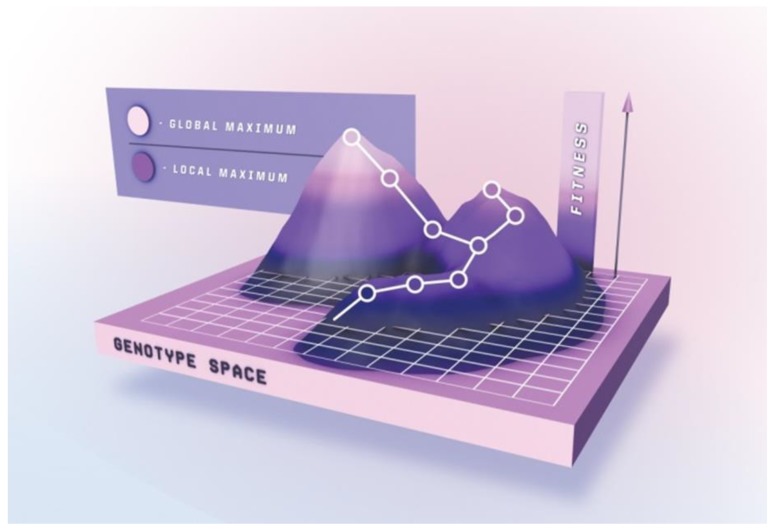
Maturation of a peptide depicted as ascent on a simplified fitness landscape. After each selection round, mutations are introduced into the enriched combinatorial library, and the next generation of peptides is screened for improved affinity and/or activity.

*Completely* randomizing even relatively short peptides would require a library size surpassing the capacities of most platforms. Sampling the complete mutational space for peptides exceeding 8–9 residues is therefore practically impossible, and gene diversification strategies only allow for generation and subsequent interrogation of a limited subset of the entire theoretical peptide population. Peptide maturation can be depicted as an ascent in a simplified fitness landscape (Figure 3) in which the x-y coordinates denote the otherwise multidimensional genotype, and the z-axis represent the peptide’s “phenotypic” traits, e.g., target affinity. Ascending towards peak activity with mutational steps is the goal of directed evolution. Beneficial mutations accumulate over several generations upon selection pressure, resulting in improved phenotype [199].

In general, library generation can be performed either through focused or random mutagenesis. The latter is usually used in the absence of structure–function relationship knowledge. In focused mutagenesis, residues previously found to be essential for peptide activity are retained (or favored over the rest of the building block set), while the others are (fully or partially) randomized. Of course, the odds that a library contains improved peptide variants are higher for those produced by focused mutagenesis. A plethora of mutagenesis methods can be used for gene diversification in library generation and we will briefly discuss them below.

### 4.1. Random Mutagenesis

Random mutagenesis based on physical and/or chemical mutagens is sufficient for traditional genome screening (gene inactivation), but it is not suitable for directed evolution due to limited mutational spectrum [200,201]. For library generation purposes, random mutagenesis can be performed in vivo in bacterial mutator strains that contain defective proofreading and repair enzymes (mutS, mutT, and mutD) [202,203,204]. Another approach in *E. coli* relies on mutagenesis plasmids (MP), which carry multiple genes for proteins affecting DNA proofreading, mismatch repair, translesion synthesis, base selection, and base excision repair, thereby enabling broad mutagenic spectra. MPs support mutation rates 322,000-fold over basal levels and are suitable for platforms based on bacterial and phage-mediated directed evolution [205]. Unfortunately, beside the library gene, mutator strains and MPs also induce deleterious mutations in host genome. In eukaryotes, this was overcome by the development of orthogonal in vivo DNA replication apparatus, which in essence utilizes plasmid–polymerase pairs, limiting mutagenesis to a cytoplasm-only event [206]. Related phenomena are also known to occur in nature (e.g., the *Bordetella bronchiseptica* bacteriophage error-prone retroelement, which selectively introduces mutations into the gene encoding the major tropism determinant (Mtd) protein on the phage tail fibers [207]) and can be exploited for creating libraries [208].

One of the most established methods for in vitro random mutagenesis is the error-prone PCR (epPCR), first described in 1989 [209]. It works by harnessing the natural error rate of low-fidelity DNA polymerases, generating point mutations during PCR amplification. However, even the faulty *Taq* DNA polymerase is not erroneous enough to be useful for constructing combinatorial libraries under standard amplification conditions. The fidelity of the reaction can be further reduced by altering the amount of bivalent cations Mn^2+^ and Mg^2+^, introduction of biased concentrations of deoxyribonucleoside triphosphates (dNTPs) [210], using mutagenic dNTP analogues [211], or adjusting elongation time and the number of cycles [212]. Random mutations can also be induced by utilizing 3’-5’ proofreading-deficient polymerases [213,214,215].

Despite its popularity, epPCR suffers from limited mutational spectrum as it inclines to transitions (A↔G or T↔C). Thus, epPCR-generated libraries are abundant in synonymous and conservative nonsynonymous mutations as a result of codon redundancy [199]. Ideally, all four transitions (AT→GC and GC→AT) and eight transversions (AT→TA, AT→CG, GC→CG, and GC→TA) would occur at equal ratios, with the desired probability, and without insertions or deletions [216]. This problem has been addressed by the sequence saturation mutagenesis (SeSaM) [217] method, which utilizes deoxyinosine, a promiscuous base-pairing nucleotide that is enzymatically inserted throughout the target gene and later changed for canonical nucleotides using standard PCR amplification of the mutated template gene. SeSaM was later improved with the introduction of SeSaM-Tv-II [218], which generates sequence space unobtainable via conventional epPCR by increasing the number of transversions. It employs a novel polymerase with increased processivity, allowing efficient read through consecutive base-pair mismatches. EpPCR has been successfully adopted for library generation in various platforms, including phage [219], *E. coli* [220], and ribosome [221] display.

Alternatively, mutagenesis can be achieved by performing isothermal rolling circle amplification (RCA) under error-prone conditions. Using a wild-type sequence as a template, this method is able to generate a random DNA mutant library, which can be directly transformed into *E. coli* without subcloning [222]. RCA was advanced further, coupling it with Kunkel mutagenesis [223] (see below). Termed “selective RCA” (sRCA), it operates by producing plasmids in *ung^-^* (uracil-DNA-glycosylase deficient) *dut^-^* (dUTP diphosphatase deficient) *E. coli* strain to introduce non-specific uridylation (dT→dU). After PCR with mutagenic primers, abasic sites are created by the uracil-DNA glycosylase in the uracil-containing template. Only mutagenized products are amplified by RCA, excluding non-mutated background sequences [224].

Although epPCR generates high mutational rates, the sequence space remains mostly untapped [225]. DNA shuffling is touted to be superior to epPCR and oligonucleotide-directed mutagenesis because it does not suffer from the possibility of introducing neutral or non-essential mutations from repeated rounds of mutagenesis [226]. DNA shuffling was the first in vitro recombination method and it involves random fragmentation of a pool of closely related dsDNA sequences and subsequent reassembly of fragments by PCR [227]. Such template switching generates a myriad of new sequences and improves library diversity by mimicking natural sexual recombination [228]. Meyer et al. [225] developed an approach where DNase I creates double-stranded breaks at the regions of interest, followed by denaturation and reannealing at homologous regions. Hybridized fragments then serve as templates and are subjected to repeated PCR rounds to form a whole array of new sequences. Improved methods were developed, eliminating the lengthy DNA fragmentation step. In the “staggered extension process” (StEP), polynucleotide sequences can be diversified through severely-abbreviated annealing/polymerase-catalyzed extension. In each cycle, growing fragments switch between different templates and anneal to them based on sequence complementarity. They then extend further and the cycle is repeated until full-length mosaic sequences are formed [229]. Another ingenious method for creating random customized peptide libraries by Fujishima et al. [230] works by shuffling short DNA blocks with dinucleotide overhangs, enabling efficient and seamless library assembly through a simple ligation process.

Currently, recombination methods are shifting from in vitro to *in vivo*. Taking advantage of the high occurrence of homologous DNA recombination events in *S. cerevisiae*, the “mutagenic organized recombination process by homologous in vivo grouping” (MORPHING) method was developed. MORPHING is a “one-pot” random mutagenesis method allowing construction of libraries with various degrees of diversity. Short DNA segments are produced by epPCR, and subsequently assembled with conserved overlapping gene fragments and the linearized plasmid by in vivo recombination upon transformation into yeast cells [231]. Another technique for assembling linear DNA fragments with homologous ends in *E. coli* is called “in vivo assembly” (IVA). IVA uses PCR amplification with primers designed to substitute, delete, or insert portions of DNA, and to simultaneously append homologous sequences at amplicon ends. Finally, it exploits recA-independent homologous recombination *in vivo*, greatly simplifying complex cloning operations. Thus, multiple simultaneous modifications (insertions, deletions, point mutations, and/or site-saturation mutagenesis) are confined to a single PCR reaction, and multi-fragment assembly (library construction) proceeds in bacteria following transformation [232].

### 4.2. Focused Mutagenesis

Effectively exploring the sequence landscape requires structural and biochemical data (from previous random mutagenesis studies), which can be leveraged to constrain genetic variation to distinct positions of the (poly)peptide, such as regions of the peptide aptamer scaffold which can endure substitutions/insertions/deletions without affecting their overall protein fold, or those peptide residues considered not absolutely essential for specific property of interest (and whose mutation might further augment peptide’s activity). Random mutagenesis results in stochastic point mutations at codons corresponding to such residues, but systematically interrogating the entire set of residues at a specific position requires a focused mutagenesis strategy. Focused libraries are typically smaller and more effective, as they only address the residues presumed to bestow the peptide with the property of interest [233].

#### 4.2.1. Enzyme-Based Approaches

Building a library of recombinant DNA constructs is a widely adopted practice accessible to virtually all laboratories, due to the ease of oligonucleotide synthesis and availability of commercial restriction enzymes and DNA ligases. The so-called oligonucleotide-directed mutagenesis enables point or multiple mutations to the target DNA sequence [234]. Normally, a mutagenic primer is designed and synthesized, subsequently elongated by Klenow fragment of DNA polymerase I, ligated into a vector by T4 DNA ligase and finally transformed into a competent *E. coli* strain. This process is long and includes multiple subcloning and ssDNA rescuing steps [235]. Several kits for site-specific mutagenesis based on mutagenic primers are commercially available. One of the systems works by applying a pair of forward and reverse complementary oligonucleotides with designed mutations. The primers are perfectly complementary to the template at 5’ and 3’ ends, but carry a changed central nucleotide sequence. A high-fidelity *Pfu* DNA polymerase is used to amplify the entire plasmid harboring the gene to be mutated, followed by the removal of the template by *Dpn*I (an endonuclease specific for methylated DNA) [236]. There are numerous adaptations of this method (reviewed by Tee and Wong [216]).

An approach termed “Kunkel mutagenesis” is commonly used for constructing libraries displayed on filamentous phage [237,238], because its genome is circular and single-stranded. In Kunkel mutagenesis, mutations are introduced with a mutagenic primer that is complementary to the circular ssDNA template. The template is propagated in an *ung^-^ dut^-^ E. coli* strain. This enzyme handicap results in the template DNA containing uracil bases in place of thymine. The template is recovered and hybridized with the primer and extended by polymerase, followed by transformation into *ung^+^ dut^+^* host cells [223]. Upon transformation, uridylated DNA template is biologically inactivated through the action of uracil glycosylase [239] of the *ung^+^ dut^+^* host, granting a strong selection advantage to the mutated strand(s) over the template.

Overlap extension PCR is another focused mutagenesis approach. First, two DNA fragments with homologous ends (and harboring desired mutation(s)) are amplified in separate PCR reactions by using 5’ complementary oligonucleotides. In a subsequent reaction, the fragments are combined; now, the overlapping 3’ ends from one of the strands of each fragment anneal and serve as “mega” primers for extension of the complementary strands. Finally, the construct is amplified with the two flanking primers [240]. Based on this strategy, the SLIM (site-directed ligase-independent mutagenesis) method, compatible with all three types of sequence modifications (insertion, deletion, and substitution), employs an inverse PCR amplification of the plasmid-embedded template by two 5’ adapter-tailed long forward and reverse primers (which include modifications) and two short forward and reverse primers (identical to the long ones but lacking the 5’ adapter sequences) in a single reaction, producing 4 distinct amplicons. Next, the amplicons are heat denatured and reannealed to yield 16 (hetero)duplexes, 4 of which are directly cloneable, forming circular DNA through ligation-independent pathway via complementary 5’ and 3’ single-stranded overhangs. All steps of the SLIM procedure are carried out in a single tube [241].

Gibson assembly is a method of combining up to 15 DNA fragments containing 20–40 bp overlaps in a single isothermal reaction. It utilizes a cocktail of three enzymes; exonuclease, DNA polymerase, and DNA ligase. The exonuclease nibbles back DNA form the 5’ end, enabling annealing of homologous DNA fragments. DNA polymerase then fills in the gaps, followed by the covalent fragment joining by the DNA ligase [242]. Applications of Gibson assembly include site-directed mutagenesis and library construction [243]. A recent adaptation, QuickLib, is a modified Gibson assembly method that has been used to generate a cyclic peptide library [244]. QuickLib uses two primers that share complementary 5’ ends; one long partially degenerate, and the other short non-degenerate, which are then used for full plasmid PCR amplification. Subsequently, a Gibson reaction is performed which circularizes the library of linear plasmids, followed by template elimination by *Dpn*I restriction.

Besides conventional enzymes involved in cumbersome digestion and ligation steps, other enzymes can be utilized for mutagenesis. In nature, lambda exonuclease aids viral DNA recombination. It progressively degrades the 5′-phosphoryl strand of a duplex DNA from 5’ to 3’, producing ssDNA and (mono)nucleotides [245]. To exploit this property, first, a PCR amplification using template ssDNA and phosphorylated primers with overlapping regions is performed. The PCR product is then treated with lambda exonuclease, generating ssDNA fragments that are subsequently annealed via overlap regions. Afterwards, Klenow fragment is employed to create dsDNA. In this manner, site-specific mutagenesis can be performed using primers that contain degenerate bases [246].

One of the most broadly used approaches for characterization of individual amino acid residues of a (poly)peptide with regards to their contribution to binding affinity or activity is the alanine-scanning mutagenesis. As the name implies, the technique is based on systematic substitution of residues with alanine, and assessing ligand’s activity in a biochemical assay. Alanine eliminates the influence of all side chain atoms beyond the beta-carbon, thus exploring the role of side chain functional groups at interrogated positions [247]. For example, a conventional single-site alanine-scanning was used to assess the contribution of individual amino acid residues of a Fc fragment binding peptide displayed on filamentous phage [67]. Since this type of approach is laborious, methods have been developed for multiple alanine substitutions in a high-throughput manner [248]. One such approach builds on the codon-based mutagenesis, analyzing multiple positions, applying split-and-mix synthesis to produce degenerate oligonucleotides (one pool for the alanine codon and another for the wild-type codon) [249]. An alternative to alanine-scanning is serine-scanning, which follows the logic that, sometimes, substitutions with the hydrophobic alanine side chains may be more detrimental to the peptide’s affinity compared to the slightly larger but hydrophilic serine side chain. Similarly, homolog-scanning (substitutions at individual positions with similar residues) may be employed with the goal of minimizing structural disruption and identifying residues essential for maintaining a function [250].

Another site-directed mutagenesis type is the cassette mutagenesis. It works by replacing a section of genetic information with an alternative, synthetic sequence—a “cassette” [251]. Different from other approaches that target short regions of a gene, this method is convenient for sequences up to 100 bp in size [252,253]. A prerequisite for this method to be practical is that the gene cassette must be flanked by two restriction sites that are complementary and unique with digest sites on the targeted vector. Restriction enzymes excise the targeted fragment from a vector that can then be replaced with DNA sequences carrying desired mutations. If a larger fragment is to be cloned, the “megaprimer” approach is applied by amplification with a series of oligonucleotides [254]. This method can also benefit from using “spiked” synthetic oligonucleotides, allowing randomization at multiple sites [255,256]. Cassette mutagenesis is based on Kunkel mutagenesis, which is time-consuming, so researchers developed an improved version termed ‘’PFunkel’’, a conflation of *Pfu* DNA polymerase and Kunkel mutagenesis, that can be performed in a day’s work [257,258]. To overcome the main constraint of site-directed mutagenesis, which is the tedious primer design, rational design techniques can be utilized to introduce desired mutations at precise positions. Researchers can leverage readily available tools such as AAscan, PCRdesign, and MutantChecker to simplify and boost the mutagenesis process [259].

#### 4.2.2. Chemical-Based Mutagenesis

Chemical-based mutations involve various chemical methods to produce desired mutants. To chemically synthesize fully randomized oligonucleotides, a mixture of nucleotides must be applied at each coupling step [260]. A calamitous problem with this strategy is the pronounced bias resulting from the uneven incorporation frequency of the 4 nucleotide building blocks due to their inherent reactivity differences, rendering statistical random mutations inaccessible. Avoiding incorporation of stop codons is practically unattainable and the system is inclined towards amino acid residues encoded by redundant codons [261]. This problem can be tackled by adjusting the mutational frequency with “spiked oligonucleotides” [255], taking into account the differences in reactivity of mononucleotides and the redundant genetic code. The essence of DNA spiking is that non-equimolar ratio of bases at targeted positions are applied during oligonucleotide synthesis, meaning each wild type nucleotide can be custom “doped”, achieving either “soft” (high incidence of a certain nucleotide) or “hard” (equal incidence of all four nucleotides) randomization, manually tuning the occurrence of certain amino acids at defined positions in the (poly)peptide chain.

Site-saturation mutagenesis seeks to achieve mutation at a maximal capacity by examining substitutions of a given residue against all possible amino acids. A fully randomized codon NNN (where N = A/C/G/T) gives rise to all possible 64 variant combinations (also known as 64-fold degeneracy) and codes for all 20 amino acids and 3 stop codons. This causes difficulties during library screening and risks enrichment of non-functional clones due to the random introduction of termination codons [262]. Operating with NNK, NNS, and NNB codons (where K = G/T, S = C/G, and B = C/G/T) minimizes the degeneracy in the third position of each codon, consequently lowering codon redundancy and the frequency of terminations [263]. However, such degenerate primers are expensive to synthesize, and using a single degenerate primer to completely eliminate codon redundancy while providing all 20 amino acids is unattainable, due to disproportional representation of certain amino acids [264,265]. Other strategies have to be employed to circumvent these constraints.

To synthesize redundancy-free mutagenic primers, mono [266], di [267], or trinucleotide phosphoramidite [268] solutions (or combinations [269]) can be used. This way, mixtures of oligonucleotides encoding all possible amino acid substitutions within a defined stretch of peptide or a limited number of amino acids (i.e., “tailored” randomization) can be synthesized. This fine-tuning gives complete control over amino acid prevalence at defined positions in the corresponding (poly)peptide sequence, achieving “soft” or “hard” randomization. With this approach, codon redundancy and stop codons are completely eliminated [261]. Another randomization strategy labeled MAX eliminates genetic redundancy by using a collection of 20 primers containing only codons for each amino acid with the highest expression frequency in *E. coli* [270]. These primers are annealed to a template strand with completely randomized codons (NNN or NNK) at the targeted position. Any misannealing is trivial, since only the ligated selection strand is amplified by a subsequent PCR. The produced random cassettes are then enzyme-digested for cloning. Further development of this strategy gave birth to an upgraded version dubbed ProxiMAX in which multiple contiguous codons are randomized in a non-degenerate manner [271]. Here, a donor blunt-end dsDNA with terminal MAX codons and an upstream *Mly*I restriction site is ligated to an acceptor blunt-end dsDNA. The product strands are amplified, analyzed, and combined at desired ratios in the next randomization cycle. After each ligation cycle, endonuclease *Mly*I is applied to remove the donor DNA strand, making only the randomized sequences available for the successive ligation cycle.

Another strategy that has been developed by Tang et al. [264] is cost-effective and uses degenerate codons to eliminate or achieve near-zero redundancy. A mixture of four codons, NDT, VMA, ATG, and TGG (where D = A/G/T, V = A/C/G, M = A/C) with a molar ratio of 12:6:1:1 at each randomized position results in an equal theoretical distribution for each of the 20 amino acids, without occurrences of stop codons. Following a similar rationale, Kille et al. [265] developed the ‘’22c-trick’’ which uses only three codons per randomized position; NDT, VHG, and TGG (where H = A/C/T), at 12:9:1 molar ratio. The name sprung from the usage of 22 unique codons, achieving near uniform amino acid distribution (i.e., 2/22 for Leu and Val, and 1/22 for each of the remaining 18 amino acids). Other sophisticated primer mixing strategies have been reported [272,273,274], although picking the best approach is mostly dependent on the size and quality of the library to be prepared, and the lab’s operating budget [275].

## 5. Conclusions

“Design, build, test, repeat” is the core philosophy of synthetic biology. Considering the intricacies of biological systems, every step of this process is potentially affected by multiple obstacles. Researchers are often put in predicaments where their progress is halted by unpredictable issues, and solving them can last even months at a time. These are not sporadic isolated events, but rather frequent and occur in virtually every field in natural sciences, let alone molecular biology. Building a peptide library is no different; it involves miscellaneous techniques and is often laborious. Recent advances are trumping bottlenecks in practically every facet of this technology. Today, new technologies enable accurately modeling peptide structure as long as 40 residues [276], and in silico design and screening using bioinformatics tools like Rosetta [277]. Furthermore, the ability to synthesize oligonucleotides adequate in size to code for potential peptide binders and assemble them in a display format of choice is now available even to labs with tight resources [275]. Alternatively, commercial peptide libraries can be purchased, which is very convenient especially for small operations with limited personnel, equipment, and know-how. Owing to automation, unprecedented parallel screening ability is now a reality, and coupled with high-throughput deep DNA sequencing [31], discovery of large numbers of novel high-affinity binders is a realistic prospect.

So, what predictions can we make for the future of peptide discovery? Surely, the convergence of new molecular strategies coupled with novel high-throughput methods and machine learning [278] will aid future bench researchers with engineering novel peptide binders. Initiating such projects could one day be as simple as running a computer program, and ordering (or synthesizing) a library—an effortless endeavor.

## Abbreviations

AAV	Adeno-associated virus
AcMNPV	*Autographa californica* multiple nuclear polyhedrosis virus
APEx	Anchored periplasmic expression
ARS	Aminoacyl-tRNA synthetase
cPTM	Chemical post-translational modifications
Cvap	Cv RNA-associating protein
DEL	DNA-encoded library
dNTP	deoxyribonucleoside triphosphate
epPCR	Error-prone PCR
FACS	Fluorescence activated cell sorting
FIT	Flexible in vitro translation
Fmoc	Fluorenylmethyloxycarbonyl
IVA	*In vivo assembly*
IVC	*In vitro* compartmentalization
MACS	Magnetic-activated cell sorting
MHC	Major histocompatibility complex
MORPHING	Mutagenic organized recombination process by homologous in vivo grouping
MP	Mutagenesis plasmid
MS	Mass spectrometry
NGS	Next-generation sequencing
OBOC	One-bead-one-compound
PERISS	intra periplasm secretion and selection system
PPI	Protein-protein interaction
PURE	Protein synthesis using recombinant elements
RaPID	Random non-standard peptide integrated discovery
RCA	Rolling circle amplification
SAR	Structure-activity relationship
SeSaM	Sequence saturation mutagenesis
SICLOPPS	Split-intein circular ligation of peptides and proteins
SLAY	Surface localized antimicrobial display
SLIP	Site-directed ligase-independent mutagenesis
SPPS	Solid-phase peptide synthesis
StEP	staggered extension process
VLP	Virus-like particle
